# neuroRAD 2022: Precision. Individualized

**DOI:** 10.1007/s00062-022-01163-0

**Published:** 2022-06-10

**Authors:** Peter Schramm, Claus Zimmer

**Affiliations:** 1 Department of Neuroradiology, Universitätsklinikum Schleswig-Holstein, Lübeck, Germany; 2 Department of Neuroradiology, Klinikum rechts der Isar, Technical University of Munich, Munich, Germany

Dear colleagues,

It gives us great pleasure to invite you to neuroRAD 2022, the 57th Annual Meeting of the German Society of Neuroradiology, which will be held in Kassel, Germany, 12–14 October 2022.

According to the current status of the pandemic situation, we can expect to be able to host the upcoming annual meeting again as a live congress in full presence after two digital formats. For this purpose, we were able to win the Kongress-Palais in the documenta city of Kassel as the venue, an extraordinary place that combines culture and innovation in equal measure and offers plenty of space for an exciting and relaxed neuroRAD.

Our congress motto “Precision. Individualized” picks up on the continuously evolving topics of patient-specific therapies in interventional neuroradiology, but also focuses on software solutions that optimize paths and times for specific diagnostics and individualized patient treatment and accelerate the dissemination of innovations. “Precision. Individualized” also reflects the increasingly individualized ways of working in our field, from teleradiology to robotics.

neuroRAD 2022 will feature lectures on current topics in interventional neuroradiology, neuro-oncology, neurodegenerative diseases, and spine in many state of the art and scientific sessions. In addition, this year’s congress will have a special focus on pediatric neuroradiology. It is a special honor and pleasure to welcome Prof. A. James Barkovich as keynote speaker on the latter topic.

Another keynote lecture will deal with robot-assisted procedures in interventional neuroradiology. The renowned expert Prof. Vitor Pereira will tell us about the different possibilities of these new technologies and his experiences with them, which we are very much looking forward to!

At neuroRAD 2022, we want to highlight the importance of neuroradiology for the medicine of the future and show which changes in diagnostics and therapy can be expected due to new developments in the field of artificial intelligence. From the perspective of a highly technologized specialty, it is important for us not to make the discussions around these new achievements too one-sided. We are therefore very excited about the keynote lecture by Prof. Alena Buyx, in which she will address the increasing penetration of medicine by artificial intelligence from an ethical perspective.

Of course, we are also continuing formats from previous years: *Fit for Neuroradiology*, the interactive continuing education for your daily routine, diagnostic cases, video cases on interventional neuroradiology and an independent lecture program for technicians and radiographers on examination techniques, important clinical pictures and professional policy topics are also an integral part of the annual meeting, as are the proven special courses. The *Forum Junge Neuroradiologie* will address current topics in continuing education and training, clinical and scientific work, and professional development.

In addition, neuroRAD 2022 will also feature the 4th German Stroke School, a special lecture and simulation course on interventional stroke treatment. And once again, the German Stroke School lecture curriculum, covering all relevant topics in interventional stroke treatment, will be free to all neuroRAD 2022 attendees. For a limited number of participants, a one and a half day hands-on training with the latest simulation techniques under the mentoring of experienced neurointerventionalists will be offered. Learn hands-on stroke care from experts in the field!

After 2 years of pandemic virtuality, we want this neuroRAD to be an open lively congress where scientific and collegial exchange takes place in a relaxed atmosphere. We look forward to your scientific contributions, which you can present to the community both in the form of plenary lectures and during moderated poster sessions. neuroRAD 2022 also offers social events that give you the opportunity to meet your friends and colleagues: for this purpose, there are also lounge areas for casual conversations as well as one or the other event you can already look forward to. Be curious …

On our website www.neurorad.de you will find further details on the congress program, abstract submission and registration for neuroRAD 2022. We look forward to your participation in the conference, as speakers, as submitters of scientific papers and cases, and last but not least as visitors.

We are very much looking forward to meeting you in Kassel in October 2022!

Yours sincerely,

*Prof. Dr. Peter Schramm, Lübeck*

Congress President

*Prof. Dr. Claus Zimmer, Munich*

President of DGNR

